# Supplementary material for: The development and implementation of a community engagement strategy to improve maternal health in southern Mozambique
Source: PLOS Glob Public Health. 2023 Jan 30;3(1):e0001106. doi: 10.1371/journal.pgph.0001106 (PMC10021229; doi:10.1371/journal.pgph.0001106)
Supplement: Attachment — Submitted filename: Response letter CE implementation_final.pdf [file pgph.0001106.s001.pdf]

cism
centro de investigação em saúde de manhiça

Hannah Tappis, DrPH, MPH
Academic Editor
PLOS Global Public Health
1265 Battery Street, Suite 200
San Francisco, CA 94111
United States

Manhiça, 31th August 2022

Subject: Submission of the Manuscript "The Development and Implementation of a Community Engagement Strategy to Improve Maternal Health in Southern Mozambique"

Dear Editor,

Please find enclosed the manuscript "The Development and Implementation of a Community Engagement Strategy to Improve Maternal Health in Southern Mozambique", by Felizarda Amosse et al, to be considered for inclusion in your journal, having each of the points brought up by the reviewers been addressed as follows:

<u>Journal Requirements:</u>

1. You indicated that you had ethical approval for your study. In your Methods section, please ensure you have also stated whether you obtained consent from parents or guardians of the minors included in the study or whether the research ethics committee or IRB specifically waived the need for their consent.
   Response: The community engagement intervention was nested in a wider randomized control trial (the CLIP trial), which was a community-wide intervention. While organizing and conducting community engagement activities, the CE team did not deliberately select individuals aged below 18. However, also in respect of the local dynamics of rural communities, mothers were allowed to bring their children (including adolescents) along with them to the meetings. These situations are typical of our rural contexts, where the majority of women cannot afford to leave their children at home while taking part in community events. We have added the following in the methods, "Written informed consent was obtained from households participating in the overall CLIP Trial, of which community engagement was a nested component. Following the CLIP protocol, adolescents in the overall Trial gave consent based on a special assent form combined with a parental or guardian consent, and adolescents who came to community engagement events that took place in CLIP intervention clusters did so with their adult family relative, therefore considered as tacit consent to their participation in the community events." Please see lines 210-215.
   .
2. Please amend your detailed online Financial Disclosure statement. This is published with the article. It must therefore be completed in full sentences and contain the exact wording you wish to be published. State the initials, alongside each funding source, of each author to receive each grant.

Response: We have revised to, "This work is part of the University of British Columbia PRE-EMPT (Pre-eclampsia/Eclampsia, Monitoring, Prevention and Treatment) initiative supported by the Bill & Melinda Gates Foundation (Grant number: OPP1017337, PvD). Following input into trial design, the Gates Foundation had no role in data collection, analysis, or interpretation, or writing of the report."

3. Please ensure that the funders and grant numbers match between the Financial Disclosure field and the Funding Information tab in your submission form. Note that the funders must be provided in the same order in both places as well
   Response: This has been checked and the two fields match. Please see response to comment 2.

4. In the online submission form, you indicated that "The datasets generated during and/or analyzed during the current study are available from the corresponding author on reasonable request." All PLOS journals now require all data underlying the findings described in their manuscript to be freely available to other researchers, either 1. In a public repository, 2. Within the manuscript itself, or 3. Uploaded as supplementary information. This policy applies to all data except where public deposition would breach compliance with the protocol approved by your research ethics board. If your data cannot be made publicly available for ethical or legal reasons (e.g., public availability would compromise patient privacy), please explain your reasons by return email and your exemption request will be escalated to the editor for approval. Your exemption request will be handled independently and will not hold up the peer review process, but will need to be resolved should your manuscript be accepted for publication. One of the Editorial team will then be in touch if there are any issues.
   Response: Thank you for the important comment. The institutional policy does not allow data sharing in these terms. We confirm that sharing demographic and the raw qualitative data revealing the statements by community members was not part of the protocol, nor were we aware of or envisioning PLOS journals new policies at the time of protocol implementation. For these reasons we did not organize the data in such a way that guarantees that identity cannot be triangulated, therefore we are not confident that confidentiality is assured. However, the data are available upon request to the CISM's institutional ethics committee (sozinho.acacio@manhica.net) and the Mozambican Ministry of Health National Bioethics Committee (jflschwalbach@gmail.com) for researchers who meet the criteria to access confidential data. Such processes would include the signature of a confidentiality agreement as well as the compliance with additional mechanisms to ensure traceability of the access and use of this particular dataset. We have revised our data availability statement accordingly to the following, "The data are available upon request to the CISM's institutional ethics committee (sozinho.acacio@manhica.net) and the Mozambican Ministry of Health National Bioethics Committee (jflschwalbach@gmail.com) for researchers who meet the criteria to access confidential data."

5. Please provide separate figure files in .tif or .eps format and ensure that all files are under our size limit of 10MB.
   Response: The figure files are provided separately in .tif format

6. The following figures have poor resolution: Figures 1, 2 and 3. Please provide higher resolution versions.
   Response: High resolution figure files have been provided.

7. All figures and supporting information files will be published under the Creative Commons Attribution License (creativecommons.org/licenses/by/4.0/). Authors retain ownership of the copyright for their article and are responsible for third-party content used in the article.

   Figure 1: please (a) provide a direct link to the base layer of the map used and ensure this is also included in the figure legend; (b) provide a link to the terms of use / license information for the base layer. We cannot publish proprietary or copyrighted maps (e.g. Google Maps, Mapquest) and the terms of use for your map base layer must be compatible with our CC-BY 4.0 license.
   If your map was obtained from a copyrighted source please amend the figure so that the base map used is from an openly available source. Alternatively, please provide explicit written permission from the copyright holder granting you the right to publish the material under our CC-BY 4.0 license.
   Please note that the following CC BY licenses are compatible with PLOS license: CC BY 4.0, CC BY 2.0 and CC BY 3.0, meanwhile such licenses as CC BY-ND 3.0 and others are not compatible due to additional restrictions.
   If you are unsure whether you can use a map or not, please do reach out and we will be able to help you. The following websites are good examples of where you can source open access or public domain maps:
   * U.S. Geological Survey (USGS) - All maps are in the public domain. (http://www.usgs.gov)
   * PlaniGlobe - All maps are published under a Creative Commons license so please cite "PlaniGlobe, http://www.planiglobe.com, CC BY 2.0" in the image credit after the caption. (http://www.planiglobe.com/?lang=enl)
   * Natural Earth - All maps are public domain. (http://www.naturalearthdata.com/about/terms-of-use/)
   Please upload any written confirmation as an 'Other' file type. It must clarify that the copyright holder understands and agrees to the terms of the CC BY 4.0 license; general permission forms that do not specify permission to publish under the CC BY 4.0 will not be accepted. Note that uploading an email confirmation is acceptable
   Response: We have removed the base layer and have used our own data. There are no copyrights associated with the data used to produce the map.

Reviewers' comments:

REVIEWER # 1

Congratulations to the authors!
This study covers a very important topic in the context of maternal mortality reduction strategy. I have very few questions for clarification:

1. In the abstract, line 31, you mention as one of the CE message, the information about the trial, and I believe related to that you mention in the Box 1, number 7, the study protocol. I am wondering what kind of aspect were discussed with the community in this regard.

Response: Thank you for the positive review. Regarding the aspects of the study protocol discussed with the community, we have added the following line as a footnote to Box 1 to clarify, "Included discussion of the PRE-EMPT study objectives, target groups to be recruited and study procedures" See line 176-177.

2. Being used a mix methodology one could expect to see more of this combination in exploring the findings to support the conclusions. In my view the manuscript describe very well the process, however I still not clear what was the final impact. There was any change on the identified barriers to community health? There was any attempt to measure them?
   Response: Thank you for this insightful comment. We have added the following to the discussion to highlight findings that combined results from both the qualitative and quantitative datasets, "Considering the quantitative and qualitative findings together, both datasets highlight the challenge of engaging husbands and partners. The descriptive statistics show the lower numbers of men who participated in engagement activities, while qualitative feedback found that some men questioned why study activities focused on pregnant women and excluded male health concerns." See lines 359-363.

REVIEWER # 3:
This paper is written clearly and succinctly overall. It provides process evaluation of community engagement activities related to a larger trial.

Title:
1. Includes the word "development" but there is little detail regarding the development of the strategy. Suggesting removing this word or adding more detail about the development of the strategy.
   Response: Thank you for the important comment. We have further elaborated on the development of the community engagement strategy. In the "Community engagement strategy development" sub-section under "methods, we added that "The feasibility study identified a number of maternal health barriers that formed topics to be addressed within our CE strategy." These included a low awareness about pregnancy danger signs, especially pre-eclampsia and eclampsia, and the late disclosure of pregnancies. Also, regarding the poor distribution of services (see response to comment 3), we also elaborated on how findings from the feasibility study helped us develop accompanying projects that was beyond the scope of the CE strategy. We added the lines, "The feasibility study also identified local contextual factors that could influence capacity for care-seeking, which could be addressed by different accompanying components of the PRE-EMPT project. Because lack of transport was identified as a major contributor to poor referrals and represented a barrier to adequate maternal healthcare access (14), a community transport strategy was also developed to leverage existing community resources within a group savings scheme. The community transport strategy is reported further in Amosse et al 2021 (15). We also consulted community members, APEs, health providers at health facilities and the Mozambican Ministry of health regarding our CE strategy to ensure it was locally feasible and appropriate. The Ministry of Health provided important inputs on potential stakeholders and target messages." Please see lines 110-129.

Abstract:

2. No specific comments, except use of the word “successful” which I’ll discuss later.
   Response: We agree with the comment raised by reviewer 3 and have changed the wording from “successful” to “effective”, as also changed in the conclusion (see response to comment 19). See line 42.

Introduction:

3. Page 6, line 56 – “suggesting sub-optimal health care seeking behaviors” – it also may suggest lack of availability of services. It would be good to discuss the availability of services briefly.
   Response: Thank you for highlighting this important issue. We have added “and poor distribution of services” with a reference (Makanga et al 2017) into the introduction. See lines 56-57. Note that organizing transport is one of the CE messages (see Box 1). Also, we have further elaborated on the community transport strategy developed in the PRE-EMPT project alongside the CE strategy: “Because lack of transport was identified as a major contributor to poor referrals and represented a barrier to adequate maternal healthcare access (14), a community transport strategy was also developed to leverage existing community resources within a group savings scheme. The community transport strategy is reported further in Amosse et al 2021 (15).” See line 121-124.

Methods:

Implementation

4. The text indicates that activities took place in three spaces: PHCS, homes of pregnant/puerperal women and in community meetings. Does the data from the “CE logs” include only the community meetings, or all three of these contacts?
   Response: CE logs were completed after all CE sessions. We have reworked the line in the manuscript to be more explicit, “CE logs were completed by facilitators after all CE sessions at the health facility, individual homes and community meetings.” Please see lines 176-177.

5. Regarding the validity of the CE logs, I can imagine they might undercount the total participation, if some logs were misplaced or facilitators forgot to record them. However, they might over count, if facilitators felt pressured to show productivity. I’d like the authors to reflect on the validity of the logs and whether anything was done to validate this data source.
   Response: Thank you for raising this important topic. We have added further detail on our quality control process to ensure validity of the logs, “CE logs were monitored by mobilizers once a week and quality checked by community engagement supervisor once a month. While collecting the logs there were opportunities to verify the completeness of the data and clarify or resolve missing data or discrepancies. Missing or questionable data was queried by the study team for follow up. Logs were entered into a REDCap database (Vanderbilt University, Nashville, United States). Data entry from paper records to the electronic database was quality checked for accuracy.” Please see lines 182-187.

6. What incentives, if any, were the facilitators given to engage in these extra activities?
   Response: Health staff and community leaders were not given incentives specifically for community engagement activities. Nurses and CHWs received some salary support as part of the overall PRE-EMPT project. For community leaders, this was considered part of their expected roles and responsibilities to support activities in their communities.

7. The analysis would benefit from the use of appropriate statistical tests and more sophisticated analysis. For example, performing appropriate statistical tests for the information presented in Table 3, to determine if the composition of participants varied by district. It would also be interesting to know if the pattern of meeting attendance varied over time.
   Response: We agree with the reviewer. Because the purpose of this manuscript is to describe the development and implementation of the community engagement strategy, we focused on descriptive statistics to describe the participants who were engaged and frequency of activities. Investigation about the impact of community engagement on clinical outcomes is a good recommendation, but beyond the scope of the current paper and will be written up in a follow up manuscript focused on this question. We have added the following statement to the limitations, "Lastly, because the purpose of this manuscript was to describe the development and implementation of the community engagement strategy, we focused on descriptive statistics to describe the process. Follow-up is needed to explore the impact of CE on clinical outcomes." See lines 371-374.

Results

8. Table 5 – Did the project have goals regarding the number of meetings for each group? **Response:** Thank you for the question. There was no target for number of meetings for each group. Our target groups were defined based on the geographic coverage. The mobilizers team went to each district of the study area and held as many meetings as possible for each target group, the intention was to reach as many people as possible belonging to different target groups. The number of meetings or participant was not defined for each neighborhood or study area.

9. Figure 2 – this should not be a pie chart since multiple topics were discussed at the same meeting.
   Response: Figure 2 has been updated as a bar graph.

10. Figure 3 – Change to a two-dimensional style of bar chart because the three-dimensional style doesn't add any information. I don't understand how to interpret this information – was the goal to have standard presentation of information across settings? Or was the content expected to be different depending on interaction with participants?
    Response: Figure 3 has been updated as 2D, with matched formatting with Figure 2.

11. Page 17, line 238 – note typo in spelling of the word "facility".
    Response: Thank you, this has been changed. See line 266.

12. Page 18, line 249 – says the facilitators received "some pushback" from men, and the quotations suggest men were critical of the project; were there other men who gave positive feedback, or was the feedback overwhelmingly negative?
    Response: We have clarified to add that it was an issue that emerged in the minority of critical comments compared to the majority of positive feedback. We added that, "While some men were critical of the project, many men were also positive about the project." See lines 281-282.

Discussion

13. The authors state that the intervention reached about 19000 people in a population of roughly 234000. This works out to just under 5 people per meeting or roughly 2.5 interactions per facilitator per week. At the outset, did they have any goals or projections regarding the level of coverage to achieve? And if so, was this the coverage/workload expected?
Response: The calculation of 5 people per encounter is explained by the fact that CHW home visits were counted as encounters and contributed the most to the overall number of meetings. Not only they out-numbered the community meetings, but also, since they usually engaged just one pregnant woman and at the most her husband or mother/mother in law, they brought down the average number of people per meeting. The mentioned workload fits with the expectations of activists' roles and responsibilities to go to the field three days a week and reserving the other two days of the week to schedule the meetings and stay at the office for planning/logistics and data processing.

14. Line 265 – Regarding the sentence that begins, "The focus on dialogue to deliver CE messaging helped to promote participant…" this seems like the authors' professional opinion rather than something they have demonstrated through their data. I think it's based on the knowledge that informed the development of the intervention. It's two ideas – one, that dialogue is better way of communicating (as opposed to more didactic messages, I suppose.) Two that having the facilitators keep records of their process would improve the intervention. I think the authors should expand this sentence into several sentences refer to the scientific literature that informed this thinking.
Response: We acknowledge and agree with reviewer 3's comment to better clarify the results from interpretations and justify the proposed benefits of discussion and facilitator record-keeping to support community engagement activities. We have reworked the line to more accurately reflect the results reported: "The focus on dialogue to deliver CE messaging helped to promote participant discussion on pre-eclampsia and eclampsia." Please see lines 295-296. Secondly, we separated the latter idea and expanded on it under the next paragraph on "lessons learned" with additional lines and references: "Responsiveness to adapt CE strategies according to community needs and their responses to various study components has been identified as a key element of effective community engagement (16). In our study, this was supported by encouraging CE facilitators' reflection on the process and key issues that emerged in each CE session, which provided a space to record feedback from communities, helped to strengthen facilitators' engagement with activities and supported monitoring and evaluation of the program. Facilitator reflections has been used in quality improvement initiatives to promote buy-in and motivation as well as provide the time and structure to reflect on potential adaptations to improve engagement (17,18). Please see lines 303-310.

15. Line 266 – typo – it should say "helped to strengthen".
Response: Thank you, we have reworked this paragraph.

16. Lines 288-293 –These lines are unclear, and it seems like the authors are introducing new information in the discussion section.
Response: We added a line to the methods that, "Activities led by nurses and CHWs focused more on health oriented topics, while CISM field staff focused more on research and socio-cultural elements." Please see lines 154-155. In the discussion, we revised the lines for greater clarity and to build off the added line in the methods, "Because nurses and CHW mostly

focused on clinical-oriented topics, the danger signs of pregnancy, especially pre-eclampsia and eclampsia, identification of skilled birth attendants and permission for women to seek medical care were most frequently discussed messages during CE meetings. The reliance of activists' on the capacity of community leaders to mobilize people for meetings and the intermittent presence of the CISM team in the field may have led to lower coverage of the other topics with socio-cultural elements." Please see lines 328-335.

17. Lines 326-331 – this information belongs more in the methods section.
    Response: We have reworked the section regarding the lines recommended for the methods section. We have moved the highlighted lines highlighted ("Facilitators were well trained, equipped with previous research experience, familiar with the community, and fluent in the local language…") to the methods section (see lines 155-159) and revised the final sentence in the study strengths paragraph to read, "Based on comprehensive training and familiarity with the communities, the research team had support of leaders at all levels, including involvement of the Mozambique Ministry of Health." See lines 381-382.

18. Line 338 – should be "activists" instead of "activist".
    Response: Thank you, this has now been changed. Please see line 390.

Conclusions:

19. Using the word "successful" to describe the intervention seems like an over reach, given that no outcomes are presented, and they don't describe any specific coverage targets or goals the project was trying to achieve.
    Response: We agree with the comment raised by reviewer 3 and have changed the wording from a "successful" to an "effective" implementation. Please see line 394.

All comments and corrections suggested by the reviewers were addressed as well as additional general comments corrections made by the editor and were left in track changes. We thank the editor and the reviewers for the extremely useful and constructive criticism on our manuscript. All authors have agreed upon the current version of the manuscript, and concur with its submission.

Thanking you in advance for your consideration to publish this manuscript in your journal. We look forward to your response.

Sincerely,

Khátia Munguambe, BSc, MSc, PhD
