# Supplementary material for: The development and implementation of a community engagement strategy to improve maternal health in southern Mozambique
Source: PLOS Glob Public Health. 2023 Jan 30;3(1):e0001106. doi: 10.1371/journal.pgph.0001106 (PMC10021229; doi:10.1371/journal.pgph.0001106)
Supplement: Attachment — Submitted filename: Response letter CE implementation_R2_ES_KM.FA.MWK final.docx [file pgph.0001106.s002.docx]

Hannah Tappis, Dr PH, MPH

Academic Editor

PLOS Global Public Health

1265 Battery Street, Suite 200

San Francisco, CA 94111

United States

Manhiça, 14^th^ December 2022

**Subject: Submission of the Manuscript “The Development and Implementation of a Community Engagement Strategy to Improve Maternal Health in Southern Mozambique”**

Dear Editor,

Please find enclosed the manuscript “The Development and Implementation of a Community Engagement Strategy to Improve Maternal Health in Southern Mozambique”, by Felizarda Amosse et al, to be considered for inclusion in your journal, having each of the points brought up by the reviewers been addressed as follows:

**Journal Requirements:**

1. You indicated that you had ethical approval for your study. In your Methods section, please ensure you have also stated whether you obtained consent from parents or guardians of the minors included in the study or whether the research ethics committee or IRB specifically waived the need for their consent.

**Response:** Thank you for the opportunity to clarify this question. We have reworked the ethical considerations paragraph to be more explicit: “Approval for this study was obtained from the Institutional Bioethics Research Boards of Centro de Investigação em Saúde da Manhiça (CISM, CIBS-CISM/038/14), the Mozambique National Bioethics for Health Committee (219/CNBS/14) and the University of British Columbia (UBC, H12-03497). Written informed consent was obtained from all households participating in the CLIP Trial, of which community engagement was a nested component. By giving consent to participate in the CLIP trail, participants also agreed for community engagement activities, which were only conducted in CLIP intervention clusters. Following the CLIP protocol (12), adolescents in the overall Trial gave assent combined with a parental or guardian consent. All CE records were anonymized.” Please see lines 217-227.

**Reviewer comments:**

**Comment # 1:** The paper lacks a structure/a frame that could help to achieve the expressed aims of the papers: to describe the development of the CE strategy and implementation of this CE intervention. I suggest setting a structure to describe the development of the CE Strategy that can be further to used to set out the implementation process also.

Table 2 sets out the findings from the feasibility study and how each informed 'community actions'. There are 5 actions in total. Later in the paper does not refer to these again, and rather speaks to activities (and it seems that activities is referring to 3 different types of meetings undertaken to impart key messages) but there is not a clear flow from the content of the CE strategy – the community actions you cite in Table 2 and the ‘community activities’ you proceed to speak to. It will be important to elicit that more clearly.

I would suggest reworking Table 2 to include the fuller activity descriptions. This revision to Table 2 will help to capture the conceptual journey taken from identifying barriers, to needs to defining the activities in the CE Strategy in consultation to determining the activities required to address each. Thereafter, the paper can describe the implementation of activities to meet each of these 5 needs (I note 5 – the transport scheme is in another paper and the table can capture that accordingly). For example:

LOCAL BARRIER: poor awareness of maternal health issues; pre-eclampsia
IDENTIFIED NEED (IN CONSULTATION WITH COMMUNITIES, CHW, MOH ETC) Develop and promote educational messages and visual materials about pregnancy warning signs and care seeking behaviours }
COMMUNITY ENGAGEMENT ACTIVITIES
1. 48 CHW engage in health promotion using purpose made materials in house visits for women and husbands with 48 CHW
2. 11 nurses engage in PHC health talks, using purpose made materials with women and community members led b
3. 5 activists/mobilizers facilitated community meetings supported by community leaders

**Response:** Thank you for the constructive feedback. We have reworked the “Community engagement strategy development” section to provide more details of the development process with a consistent structure between the manuscript text and table 2 (Please see lines 120-142). I

Further, we have revised table 2 as recommended. The updated table 2 has been renamed “Overview of the community engagement development and implementation plan” to better describe the CE strategy structure and a third column was added to briefly describe implementation of community engagement activities linked to the earlier described structure.

**Comment # 2:** Provide more depth in the description of implementation I would suggest that you extend your analysis to breakdown the numbers you share (In table 4 and 5) further in order to provide a clearer depiction of the implementation of the activities /meetings
• All 'meetings' are aggregated and there is no differentiation explained across the different types of meetings. Details are provided that there are 3 different spaces/types of meetings; 16 nurses at PHC, 48 CHW in home visits and 5 activist/mobiliser led community meetings. However, these are lumped together as ‘meeting’ in the tables, analysis and discussion.  Can you include a breakdown in the numbers per the type of meeting? 19000 'meetings' is too aggregated and denies an individual analysis of the different activities/meetings.

Currently the analysis does not break down by meeting types and the numbers of participants reached through the different types of meetings, and therefore misses the opportunity to drill down to describe the implementation of these as individual activities. For example - these appears to be zero (or close to zero) reporting on the number of household visits, if they were repeated or one house visit only per household, the participants in the household visits, and feedback during individual house visits with CHW which is unfortunate as this could identify if there are differences in the reflections of individuals when in their private sphere rather than a public sphere.
Stakeholder reflections from Line 247 – appears to represent the reflections form the group formats only.
The high level of aggregation of the presentation of the data means we cannot tell did husbands attend only in households or also in community meetings and PHC talks, which activity/meetings did leaders attend etc.

**Response:** Thank you for suggesting this extension of our analyses. We agree that this would be very interesting but unfortunately, we had not designed the community engagement program to investigate between different methods, but rather the combined efforts of multiple CE approaches within a complex intervention. We acknowledge this as a limitation in the analysis (line 394 – 399).

**Comment # 3:** How the participants are counted. 19169 - Is there double counting of those participants that attended recurring meetings, or recurring house visits? This is not clear. If the data that is set out in Table 3 is for individual participants that should be specified.

• Did participants return to recurring group meetings and was that captured and analysed? Repeated attendance could be an indicator of appreciation for the CE sessions.

**Response:** Thank you for the opportunity to clarify this. Participants were counted at each meeting. Recurring engagement was intended to reinforce key messages and convey continued support and mobilization. Unfortunately, because of the anonymization process, we did not capture names thus cannot account for repeat participants. We agree with the points raised by reviewer 3 and have added the following to the manuscript as a limitation: “While reoccurring participant engagement was encouraged to reinforce key messages and repeated attendance could suggest appreciation of continued awareness raising and mobilization efforts, the anonymization process of CE logs in which names were not collected limits assessment of repeated attendance.” Please see lines 392-395.

**Comment # 4:** There is 0.1% leader involvement overall. Was there a leader at all community meetings? Or at x% of public health talks etc. Were they involved in other activities?

**Response:** Though there was a low proportion of meetings targeting community leaders (0.1%) this proportion was equivalent to six meetings, roughly reflecting the numbers of administrative posts. Meetings with leaders had great impact in the community, as they served to sensitize leaders about issues that were the focus of the CE strategy, and also to obtain their support in implementing all the other CE activities. As gatekeepers, they were mainly involved in the community meetings as organizers or co-facilitators, when needed. They did not participated in the public health talks or APEs visits. We have added the following to the manuscript to clarify, “Though the numbers of meetings with community leaders represented a small proportion of overall CE meetings (0.1%), they were engaged in an impactful role in community meetings as gatekeepers and opinion leaders”. Please see lines 253-255. **Comment # 5:** For husbands – 0.9% reported overall. There is an explanation that husbands are working away so participation is low. But there is no description of the strategy devised during the Community Engagement Strategy development, and course correction during implementation to try to improve health promotion to husbands, as the key decision maker.

**Response:** We agree that engaging men was a gap and have identified this as an area for future reflection to better engage them. However, we would like to clarify that there were 105 meetings (2,5% of all meetings) with husbands/partners alone, accounted for 2.5% (105 meetings), and 36 meetings (0.9 % of all meetings) where men participated with their wives/female partners. An extremely low proportion of meetings with men was detected half way through the implementation. Therefore, the team made an effort to increase the frequency of meetings with men, and this is what resulted in the final numbers of meetings with men. However, we acknowledge that men still represented a small overall proportion of participants, which we hypothesize may be due to competing demands such as men working outside the local community. We have added the following line to invite further reflection to improve health promotion to husbands, “Future community engagement initiatives should not only invite men to maternal health discussions, but also identify barriers to their participation and reflect on best practices to improve their participation.” Please see lines 379-381.

**Comment # 6:** Use of community terms, definitions and outdated references.
The references used to define CE and the critical need for more research are from 2002 and 1991. The literature has developed substantially over the past 10 years in particular. I suggest you use a more up-to-date definition and call for action - delete reference 10 which undermines the strength of this paragraph (this is a book review 1 page from 1991 - better to go straight to WHO /UNICEF documents).
1.     Community engagement; a health promotion guide for universal health coverage in the hands of people. Geneva. World Health Organization 2020.
2.     WHO Recommendations on health promotion interventions for maternal and newborn health
• Also, the literature on community engagement has used different terminology over time and as a result there are multiple terms in use and the terms are used interchangeable; community engagement, community participation, community mobilisation etc. Three terms are used in this paper, interchangeably. In order to avoid adding to the confusion in the literature, I would suggest that to stick with the use of one term – community engagement and define that.

• CE has many definitions, and the scope can entail health promotion, health education, citizen involvement in quality and coverage improvement through M&E activities, collaboration in health facility management, community health, monitoring, dialogues with health authorities on needs etc. The purpose of the activities set out in this paper is engaging community members firstly in health promotion and awareness raising on danger signs, how to seek referral. The further activity on developing a group saving scheme is not described and I understand this is for another paper. Therefore, I would suggest you draw on an up-to-date definition that is tailor-made for community engagement for the health promotion activities that this paper is focused on: such as WHO Recommendations on health promotion interventions for maternal and newborn health (2015).

**Response:** Thank you for the thoughtful comments, which have strengthened the manuscript. To address comment 6:

a) We have updated references to recent WHO publications:

- World Health Organization. Community engagement: a health promotion guide for universal health coverage in the hands of the people. Geneva: WHO; 2020.
- World Health Organization. WHO recommendations on health promotion interventions for maternal and newborn health. Geneva: WHO; 2015.
- World Health Organization. WHO community engagement framework for quality, people-centred and resilient health services. Geneva: WHO; 2017.

b) We removed the previous outdated reference and reworked the lines to reflect current WHO statements on CE: “Consequently, the World Health Organization (WHO) recognizes the importance of engaging communities in promoting health and describes CE as vital to linking health problems to appropriate health promotion actions (10). The WHO highlights that CE can improve maternal and newborn health behaviours, increase use of skilled care and increase household and community support for maternal and newborn health (11).” Please see lines 71-77.
c) In alignment with the current WHO definition, we defined CE as “a process of developing relationships that enable stakeholders to work together to address health-related issues and promote well-being to achieve positive health impact and outcomes” Please see lines 107-109.
d) We agree with reviewer 3’s comment that “community participation” and “community engagement” are not synonymous. We have gone through the manuscript to update all mentions to “community engagement”, which is defined using the current WHO definition. Instead of using the terms interchangeably, we clarified that “CE is a process that encourages community participation in activities to improve population health and/or reduce health inequalities.” Please see lines 105-107.

**Comment # 7**: There is little analysis provided on the views and feedback of the implementers themselves during the implementation or description of how this resulted in course correction, changes, how any challenges faced were reviewed and overcome. Line 301: Highlights the value of facilitator reflection– but there is no description or examples provided of the content the facilitators reflections - the nurses and CHW in particular? It would be good to share both positive and negative reflections that arose during implementation. What were the challenges and disaggregate these such as for the challenges the nurses faced at the PHC, the CHW on house visits etc., was there a process followed for implementers to reflect, tweak the activity /change course etc during implementation?

**Response:** Thank you for highlighting this as an issue. For our community engagement strategy, a monitoring system was developed consisting of community engagement logs described in the methods section. There was no additional data collection (for instance through interviews with target groups and facilitators) to directly capture their views and experience on the process. All CE facilitators (nurses, CHWs, activists and mobilizers) completed CE logs after each session, which formed the dataset for our analyses. The log contained a space for feedback annotations but this feedback only referred to participants comments and not personal feedback of the implementers on their own perspectives of implementation. This is due to how CE facilitators were trained to use the CE logs and feedback section to highlight community perspectives, which we have described in our results. The format of CE sessions was not modified during the course of implementation but CE facilitators regularly adapted the strategy to messaging needs of different communities and also to the need to prioritize specific target groups, (for example men) and specific geographic areas with low coverage of meetings or topics. This is shown in Figure 3 as signs and symptoms of pregnancy complications was more widespread in Manhiça district, while permissions for care seeking was more widely addressed in Bilene-Macia and Chibuto districts for example.

**Comment # 8:** The paper notes that facilitators (chiefly the nurses and CHW) reported the activities were well received by the community. This is a key finding. This could be bias due to the facilitator interests to appear to a good job etc – and it would be better if there was further content analysis undertaken such as on evaluation interviews with participants, community leaders, CHW, nurses to substantiate the finding.  As it is - it would be good to qualify that statement to be clear that that finding is based on the personal views of facilitators that the activities were well received.

**Response:** Thank you for highlighting this issue. We previously highlighted sourcing facilitator feedback was a limitation of the study and have reworked the line to more explicitly the risk of bias. The line has been updated to: “qualitative data was sourced from CE facilitator feedback thus there may be some bias to reporting positive results.”
 
**Comment # 9:** Overall need to check for typos – for example.

Abstract: 2107 - 2017. Again in Line 28

Line 53 – contributes

Line 54 – services

Line 61 – suggest change management of mortality to reduction of maternal and infant mortality.

**Response:** Thank you. These have now been corrected and suggestions accepted. We have gone through the manuscript to check for grammar and flow.

All comments and corrections suggested by the reviewers were addressed as well as additional general comments corrections made by the editor and were left in track changes. We thank the editor and the reviewers for the extremely useful and constructive criticism on our manuscript. All authors have agreed upon the current version of the manuscript, and concur with its submission.

Thanking you in advance for your consideration to publish this manuscript in your journal. We look forward to your response.

Sincerely,

Khátia Munguambe, BSc, MSc, PhD
